# Direct Lp(a)-C measurements provide evidence for Apo(a) isoform-dependent cholesterol composition of Lp(a)

**DOI:** 10.1016/j.jlr.2026.101051

**Published:** 2026-04-27

**Authors:** Sotirios Tsimikas, Santica M. Marcovina

**Affiliations:** 1 Vascular Medicine Program, Division of Cardiology, University of California San Diego, La Jolla, California, USA; 2 Medpace Reference Laboratories, Cincinnati, Ohio, USA

**Keywords:** cholesterol, low density lipoprotein, lipoprotein(a), cardiovascular, diagnosis, therapy, isoforms, apolipoprotein(a), direct Lp(a)-C

## Abstract

Lipoprotein(a) [Lp(a)] carries cholesterol [Lp(a)-C], yet the cholesterol composition of Lp(a) particles and its relationship to apolipoprotein(a) [apo(a)] isoform size remains incompletely defined. Prior estimates of Lp(a)-C have relied on fixed-percentage assumptions rather than direct biochemical measurement, limiting insight into particle-level heterogeneity. We developed a direct immunocapture assay using the monoclonal antibody LPA4 to quantify Lp(a)-C in plasma and applied it to 94 individuals spanning a wide range of Lp(a) concentrations and apo(a) isoform sizes. Lp(a)-C was strongly correlated with Lp(a) molar concentration (R = 0.925, *P* < 0.001) and inversely associated with apo(a) isoform size (R = −0.745, *P* < 0.001). Across tertiles of the predominant apo(a) isoform size, smaller isoforms (12–17 KIV repeats) had higher Lp(a)-C (8.3 ± 4.3 mg/dl; 11.0 ± 3.4%), mid-range isoforms (18–23 KIV) were intermediate (5.0 ± 3.2 mg/dl), whereas larger isoforms (>24 KIV) showed lower Lp(a)-C (3.0 ± 1.5 mg/dl) (*P* < 0.001), normalized to plasma volume (not particle concentration). In contrast, particle-normalized metrics demonstrated the opposite pattern: both the Lp(a)-C/Lp(a) molar ratio and Lp(a)-C/Lp(a)-apoB mass ratio increased progressively with apo(a) isoform size (*P* < 0.001), indicating greater cholesterol content per Lp(a) particle among larger isoforms. These findings demonstrate a dissociation between circulating Lp(a)-C concentration, which primarily reflects particle number, and cholesterol content per particle, which varies systematically with apo(a) isoform size. Direct measurement of Lp(a)-C identifies compositional heterogeneity not captured by conventional estimation methods and may provide a framework for future studies of isoform-dependent variation in Lp(a) structure and function.

Lipoprotein(a) [Lp(a)] is a genetically determined, independent risk factor for atherosclerotic cardiovascular disease, promoting inflammation, thrombosis, and atherogenesis (1, 2). Although circulating Lp(a) concentration is routinely quantified by immunoassays and results expressed in nmol/L reflects particle number (3), the lipid composition of Lp(a) particles themselves is less well studied (4). The lipid core of Lp(a) is enriched in cholesteryl esters and triglycerides, while unesterified cholesterol resides predominantly in the surface monolayer. The total cholesterol carried by Lp(a) [Lp(a)-C] has not been well characterized across individuals (5, 6, 7).

This uncertainty is important because apo(a) isoform size, determined by variable kringle IV type 2 (KIV_2_) repeat number in apo(a), shows a well-established inverse relationship with circulating Lp(a) levels (8). The cholesterol content carried by Lp(a) [Lp(a)-C] has not been systematically characterized across individuals. Biochemical studies using ultracentrifugation to separate lipoprotein particles suggested potential isoform-related differences (9, 10), but the methods used may introduce variability due to recovery efficiency and particle redistribution and did not directly quantify cholesterol on native Lp(a) particles.

To overcome these limitations, we recently developed a direct immunocapture assay for Lp(a)-C using the monoclonal antibody LPA4, which selectively isolates native Lp(a) without cross-reactivity to apoB or plasminogen (7, 11, 12, 13). In the present study, we applied this assay to plasma from human donors to define the in vivo relationship between Lp(a)-C, Lp(a) concentration, and apo(a) isoform size. We hypothesized that the cholesterol content of Lp(a) particles varies systematically with apo(a) isoform size and that direct measurement of Lp(a)-C would reveal isoform-dependent differences not captured by particle number alone.

## Methods

### Human subjects

Samples were selected based on availability of prior Lp(a), LDL-C, and apo(a) isoform measurements and sufficient plasma volume, rather than to represent a population-based cohort. The sample size was informed by prior analytical validation studies and designed to support correlation analyses and tertile-based comparisons across apo(a) isoform groups. The target sample size was based on prior pilot data (7) indicating that ∼30 subjects provided robust analytic performance for direct Lp(a)-C, the desire to support Spearman correlations and tertile-based comparisons across three apo(a) isoform groups with reasonable precision, and practical constraints on apo(a) isoform phenotyping and direct Lp(a)-C throughput in a CLIA-certified laboratory setting.

### Direct Lp(a)-C method

The direct Lp(a)-C method was previously described (7) and its analytical validation was performed in a CLIA certified laboratory (13). The method is based on a direct immunocapture ELISA for quantifying Lp(a)-C in human plasma using an apolipoprotein(a)-specific monoclonal antibody (LPA4) (14) coupled to magnetic beads.

### Determination of Lp(a) molar concentrations, apo(a) isoforms, and laboratory variables

Lp(a) particle numbers in nmol/L were measured using Roche Tina-quant Gen. 2 kit on a Roche c502 analyzer (3). Each of the five standards used to calibrate the assay is formed by plasma pools with apo(a) isoform size ranging from large to small and Lp(a) concentrations from low to high. The target values to each standard were independently assigned against the WHO/IFCC SRM-2B reference material, thus resulting in minimized impact of apo(a) isoform size-generated bias. The Roche Tina-quant Lipoprotein(a) Gen.2 molar assay has been shown to agree closely with the apo(a) size-insensitive Marcovina reference ELISA method, with Deming regression slope 1.02 (95% CI 0.99–1.06), intercept 0.7 nmol/L (95% CI -1.0 to 2.4 nmol/L), and R = 0.99 over 8.7–234 nmol/L (FDA 510(k) K241220). (U.S. Food and Drug Administration. 510(k) Substantial Equivalence Determination K241220: Tina-quant Lipoprotein(a) Gen.2 Molarity Assay. Silver Spring, MD: Center for Devices and Radiological Health; 2024. Available at: https://www.accessdata.fda.gov/cdrh_docs/reviews/K241220.pdf).

Apo(a) isoform size was determined by an in-house developed highly sensitive SDS-agarose gel electrophoresis method calibrated with five plasma samples with a defined number of KIV determined by pulsed-field gel electrophoresis on the DNA of the same donors as the plasma samples (15).

The predominant apo(a) isoform was defined as the band with the highest integrated optical density by densitometry. When band intensities were similar, the smaller isoform was designated predominant. The minor isoform was defined as the second band, typically corresponding to the allele with a larger KIV_2_ repeat number.

Direct LDL-C concentration was determined by a homogeneous enzymatic method using Roche reagent and calibrator on a Beckman Coulter analyzer. Total cholesterol was determined using Beckman reagent on a Beckman Coulter analyzer.

### Conversion of Lp(a)-C to molar units and derivation of the Lp(a)-C/Lp(a) molar ratio

Lp(a)-C concentrations (mg/dl) were converted to molar units (nmol/L) by dividing the mass concentration by the molecular weight of cholesterol (386.65 g/mol) and multiplying by the unit conversion factor. Thus:

$$\text{Lp}\left(a\right)-C\;\left(\text{nmol}/L\right)=\text{Lp}\left(a\right)-C\;\left(\text{mg}/\text{dL}\;X\;10\right)\;\;/\;386.65\;g/\text{mole}\times 10^{6}$$

The molar ratio of Lp(a)-C (nmol/L to total Lp(a) (nmol/L)) was then calculated as:

MolarratioLp(a)−C/Lp(a)=Lp(a)−C(nmol/L)/Lp(a)(nmol/L)

This ratio reflects the average cholesterol content per Lp(a) particle.

### Determination of the Lp(a)-C/Lp(a)-apoB ratio

Because each Lp(a) particle contains one molecule of apoB-100, analogous to LDL, we calculated the Lp(a)-C/Lp(a)-apoB ratio as an index of cholesterol content per particle. In those studies, the ratios ranged from approximately 1.2 to 1.6 with ratios <1.2 indicating cholesterol-depleted, triglyceride-rich particles characteristic of the small dense LDL (pattern B) phenotype and ratios >1.6 representing larger, cholesterol-rich LDL particles (pattern A) (16, 17, 18).

Using an analogous approach, we calculated the Lp(a)-C/Lp(a)-apoB ratio to evaluate whether the cholesterol content of Lp(a) particles varies according to apo(a) isoform size. A uniform ratio across isoforms would imply relatively constant lipid composition and support the use of fixed-factor estimation formulas for calculating Lp(a)-C. Conversely, systematic variation in the ratio would indicate that calculating Lp(a)-C assuming a constant conversion factor is inaccurate and that an empirical measurement is required.

Lp(a) molar concentration is determined by measuring its apo(a) component. Because each Lp(a) particle contains one molecule of apo(a) and one of apoB-100, the Lp(a)-apoB molar concentration is, by definition, identical to the Lp(a) molar concentration. To express Lp(a)-apoB in mg/dl, Lp(a) molar concentrations (nmol/L) were divided by 19.49, since 1 mg/dl of apoB-100 corresponds to 19.49 nmol/L, based on the predicted nonglycosylated molecular weight of apoB-100 (513 kDa) derived from the canonical apoB-100 sequence (UniProt P04114; NCBI NP_000375.2) (19, 20).

The ratio was then calculated as:

Lp(a)−C/Lp(a)−apoBratio=Lp(a)−C(mg/dl)/Lp(a)−apoB(mg/dl)

This ratio is intended as an index of the relationship between Lp(a)-C and Lp(a) particle concentration and may provide indirect insight into variation in cholesterol content per particle, although this interpretation requires validation using purified Lp(a) preparations. Because this ratio incorporates measurements from different assays and does not directly isolate Lp(a)-associated apoB, it should be interpreted as an exploratory metric rather than a definitive measure of Lp(a) particle composition.

### Statistical analyses

Continuous variables are reported as mean ± SD or median (IQR) according to distribution. Correlations between Lp(a)-C and lipid parameters were assessed using Spearman's rho coefficients. Group comparisons across apo(a) isoform tertiles were performed using one-way ANOVA with Bonferroni correction. Normality was verified by the Shapiro-Wilk test. Statistical significance was defined as *P* < 0.05 (two-sided). Analyses were conducted in SPSS v29.0 (IBM, Armonk, NY).

## Results

### Range of Lp(a)-C values in human subjects

In this cohort of 94 individuals, Lp(a) concentrations demonstrated wide variability among individuals, ranging from 9 to 339 nmol/L, with a mean of 93.7 nmol/L (Table 1). Direct Lp(a)-C averaged 5.4 mg/dl (range 0.8–20.7 mg/dl), accounting for approximately 2.9% of the total circulating cholesterol.

**Table 1:** Lp(a) molar concentration, predominant, and minor apo(a) isoform size and measured and derived laboratory variables in study subjects

Variable	# Subjects	Mean (SD)	Median (IQR)	Minimum	Maximum
Lp(a), nmol/L	94	93.7 (76.0)	82.6 (29.5–130.9)	9	339
Lp(a)-C, mg/dL	94	5.4 (3.8)	4.4 (2.5–6.8)	0.8	20.7
LDL-C, mg/dL	91^a^	103.5 (24.8)	104.1 (86.1–121.9)	39	157.1
Total cholesterol	91^a^	177.5 (28.4)	176.8 (157.0–196.3)	108	238.9
% Lp(a)-C of total cholesterol	91^a^	2.9 (2.0)	2.6 (1.5–3.7)	0.6	12.3
Lp(a)-apoB, mg/dL	94	4.8 (3.9)	4.1 (1.5–6.8)	0.4	17.4
Predominant isoform, #KIV repeats	94	20.6 (4.5)	19 (17–25)	12	29
Minor isoform, #KIV repeats	89^b^	27.5 (5.3)	28 (24–31)	17	39
Lp(a)-C/Lp(a) molar ratio	94	1948 (956)	1,679 (1,228–2,277)	803	4,847
Lp(a)-C/Lp(a)-apoB ratio	94	1.47 (0.72)	1.27 (0.93–1.72)	0.6	3.7

Lp(a)-C/Lp(a) molar ratio is expressed as nmol cholesterol per nmol Lp(a) particle.

IQR, Interquartile range.

a Sample quantity was not sufficient for determination in all subjects.

b subjects did not express a second isoform.

Apo(a) isoform sizes displayed the expected broad variability driven by differences in KIV_2_ repeat number. The predominant isoform ranged from 12 to 29 KIV repeats, reflecting the smaller isoforms typically associated with higher Lp(a) concentration. The second isoform, present in 89 out of the 94 individuals, ranged from 17 to 39 KIV repeats and represents the lower-expressed allele, which typically corresponds to the larger apo(a) isoform.

Derived compositional indices demonstrated additional heterogeneity: the Lp(a)-C/Lp(a) molar ratio averaged 1,948 (range 803-4,847), and the Lp(a)-C/Lp(a)-apoB ratio averaged 1.47 (range 0.6–3.7), highlighting high variability in Lp(a) lipid and apoB content across individuals (Table 1).

### Association of the predominant apo(a) isoform, Lp(a)-C, Lp(a) molar concentration, and derived Lp(a) ratios

Spearman analyses demonstrated a strong positive correlation of direct Lp(a)-C with Lp(a) molar concentration (R = 0.925, *P* < 0.001) and moderate negative correlations with the predominant apo(a) isoform (R = −0.675, *P* < 0.001). Lp(a)-C also correlated negatively with the Lp(a)-C/Lp(a) molar ratio (R = −0.515, *P*< 0.001). (Table 2).

**Table 2:** Spearman rho coefficients and *P*-values for lipid and lipoprotein variables

Variable	Lp(a) molar conc	Predominant isoform	Minor isoform	Lp(a)-C/Lp(a) molar ratio
Lp(a)-C	0.925^b^	−0.675^b^	−0.329^a^	−0.515^b^
Lp(a) molar conc		−0.745^b^	−0.403^b^	−0.770^b^
Predominant isoform			0.454^b^	0.617^b^
Minor isoform				0.411^b^

a *P* < 0.05.

b *P* < 0.001.

Lp(a) molar concentration showed strong negative correlations with the predominant isoform (R = −0.745, *P* < 0.001) and the Lp(a)-C/Lp(a) ratio (R = −0.770, *P* < 0.001). The predominant isoform was positively correlated with the second isoform (R = 0.454, *P* < 0.001) and showed significant positive associations with the Lp(a)-C/Lp(a) ratio (R = 0.617, *P* < 0.001).

**Fig. 1:**
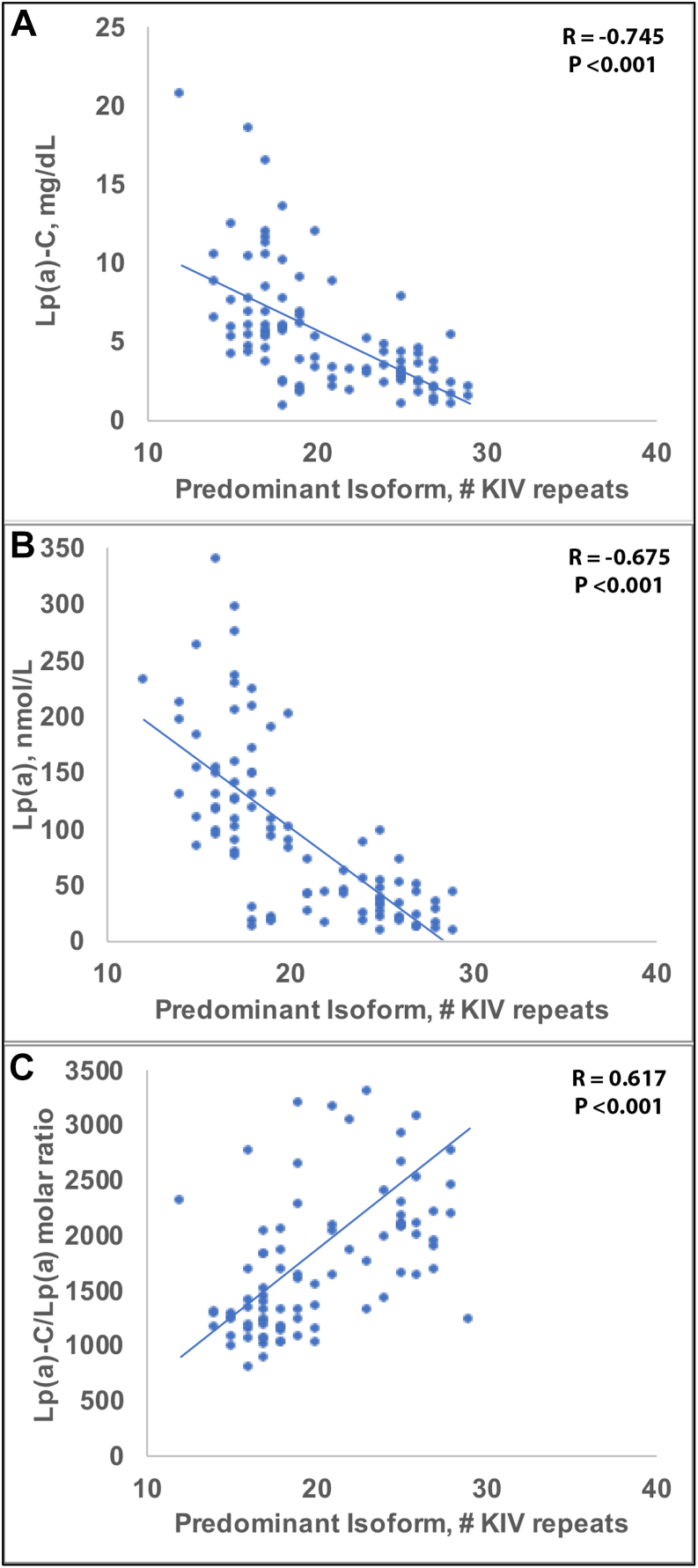
Relationship of predominant apo(a) isoform size to Lp(a) parameters. Scatterplots show Spearman correlations between predominant apo(a) isoform KIV repeat number and (A) Lp(a)-C, (B) Lp(a) molar concentration, and (C) the Lp(a)-C/Lp(a) molar ratio.

The key relationships of the predominant apo(a) isoforms to Lp(a)-C, Lp(a) molar concentration, Lp(a)-C/Lp(a) molar ratio are shown in Fig. 1A–C and for Lp(a)-C/Lp(a)-apoB ratio in Supplemental Fig. S1.

### Association of the minor apo(a) isoform, Lp(a)-C, Lp(a) molar concentration, and derived Lp(a) ratios

**Fig. 2:**
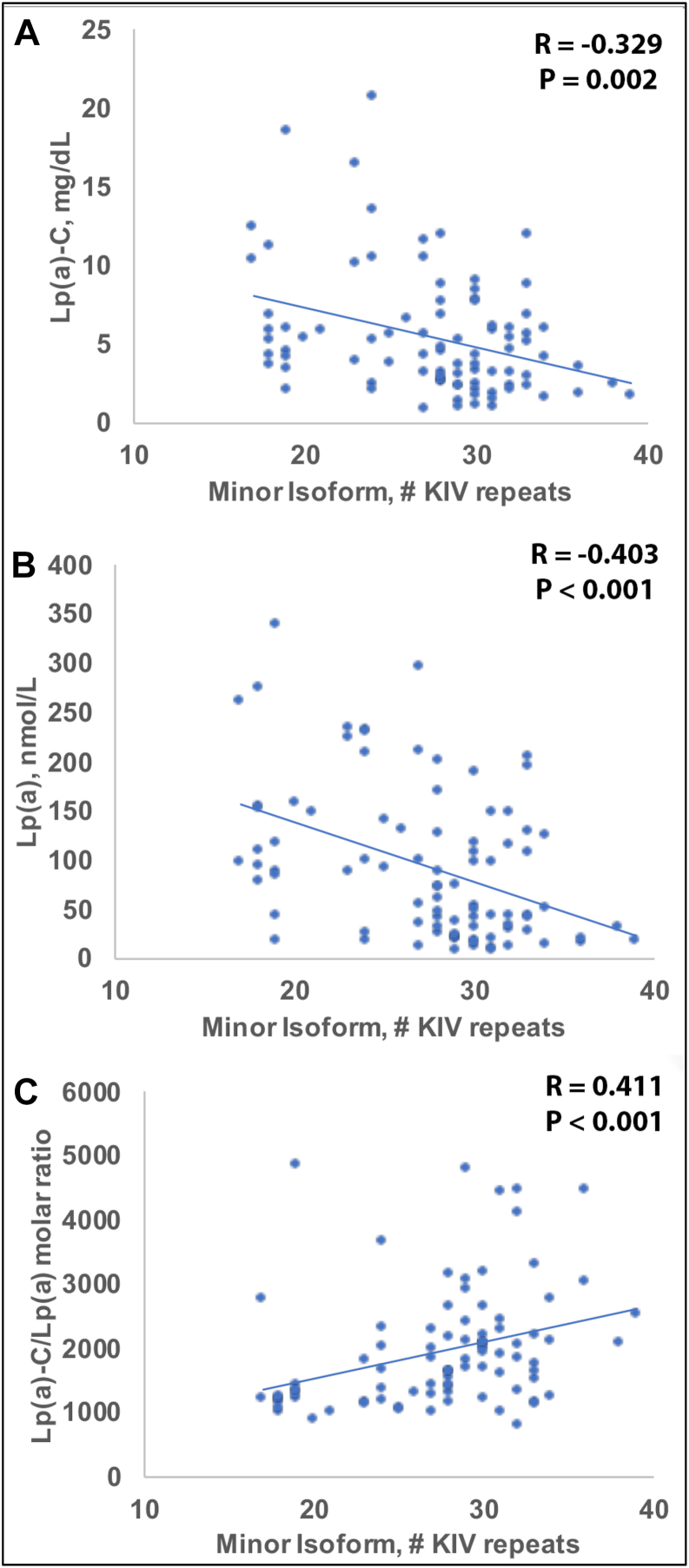
Relationship of minor apo(a) isoform size to Lp(a) parameters. Scatterplots show Spearman correlations between minor apo(a) isoform KIV repeat number and (A) Lp(a)-C, (B) Lp(a) molar concentration, and (C) the Lp(a)-C/Lp(a) molar ratio.

The relationship of the minor apo(a) isoform with Lp(a)-related variables was similar to the predominant isoform, but the associations were more modest. Spearman analyses demonstrated a moderate negative correlation of Lp(a)-C with the minor isoform (R = −0.329, *P* < 0.01), and a negative correlation with Lp(a) molar concentration showed (R = −0.403, *P* < 0.001) (Fig. 2A, B).

Positive correlations were observed between the Lp(a)-C/Lp(a) molar ratio and the size of the minor isoform (R = 0.411, *P* < 0.001) (Supplemental Fig. S2).

### Relationship of predominant isoform to Lp(a)-C, molar ratio of Lp(a)-C/Lp(a), molar ratio of Lp(a)-C/Lp(a)-apoB

Across tertiles of the predominant apo(a) isoform, all Lp(a)-C-related measures showed a graded association across isoform tertiles (Table 3). Individuals with the smallest isoforms (12–17 KIV repeats) had the highest Lp(a)-C concentrations (mean 8.34 ± 4.27 mg/dl), whereas those with the largest isoforms (24–29 KIV) had the lowest levels (2.97 ± 1.45 mg/dl; *P* < 0.001). In contrast, indices of particle-level cholesterol content showed the opposite trend. Both the Lp(a)-C/Lp(a) molar ratio and the Lp(a)-C/Lp(a)-apoB ratio increased stepwise across isoform tertiles, rising from 1,361 ± 419 to 2,650 ± 1,065 nmol/mmol for the Lp(a)-C/Lp(a) molar ratio and from 1.03 ± 0.32 to 2.00 ± 0.80 for the Lp(a)-C/Lp(a)-apoB ratio (both *P*< 0.001, Table 3).

**Table 3:** Relationship of the tertile group of the predominant isoform to Lp(a)-C, molar ratio of Lp(a)-C/Lp(a), molar ratio of Lp(a)-C/Lp(a)-apoB, and % Lp(a)-C of Lp(a) mass

Percentile group of predominant isoform (range)	Lp(a)-C (mg/dl)	Molar ratio Lp(a)-C/Lp(a)	Mass ratio Lp(a)-C/Lp(a)-apoB
1 (Lowest) (12-17 KIV)			
Mean (SD)	8.34 (4.27)	1,361 (419)	1.03 (0.32)
Median [IQR]	6.79 [3.7–20.7]	1,242 [803–2,761]	0.94 (0.81–1.08)
2 (Middle) (18-23 KIV)			
Mean (SD)	5.01 (3.16)	1811 (764)	1.36 (0.58)
Median [IQR]	3.87 [0.8–13.5]	1,627 [1,017–3,645]	1.23 (0.88–1.56)
3 (Highest) (24-29 KIV)			
Mean (SD)	2.97 (1.45)	2,650 (1,065)	2.00 (0.80)
Median [IQR]	2.70 [1.1–7.8]	2,193 [1,230–4,847]	1.65 (1.50–2.29)
*P*-value (ANOVA)	<0.001	<0.001	<0.001

### Relationship of tertiles of the predominant and minor isoform to Lp(a)-C/Lp(a) molar ratio and Lp(a)-C/Lp(a)-apoB ratio

**Fig. 3:**
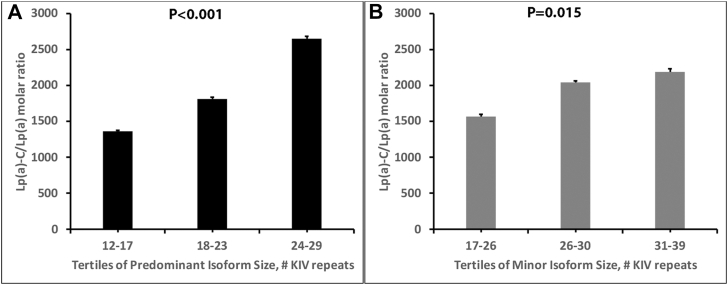
Lp(a) composition across tertiles of apo(a) isoform size. (A–B) Lp(a)-C/Lp(a) molar ratios across tertiles of the predominant (A) and minor (B) apo(a) isoforms.

Across tertiles of apo(a) isoform size, both Lp(a)-C-derived compositional indices increased stepwise. For the predominant isoform, the Lp(a)-C/Lp(a) molar ratio rose progressively across tertiles of increasing KIV repeat number (*P* < 0.001; Fig. 3A), and the same pattern was observed for the Lp(a)-C/Lp(a)-apoB ratio (*P* < 0.001; Fig. 3C). For the minor isoform, tertile analyses likewise demonstrated modest but statistically significant increases in the Lp(a)-C/Lp(a) molar ratio (*P* = 0.015; Fig. 3B) and in the Lp(a)-C/Lp(a)-apoB ratio (*P* = 0.015; Supplemental Fig. S3).

## Discussion

This study suggests that apo(a) isoform size is associated with heterogeneity in the cholesterol composition of Lp(a) particles. Using a recently validated direct Lp(a)-C immunocapture assay, we show that individuals with smaller apo(a) isoforms have higher circulating total Lp(a) and total Lp(a)-C concentrations, whereas those with larger isoforms exhibit lower absolute Lp(a)-C but greater cholesterol content per particle. These findings demonstrate a dissociation between circulating Lp(a)-C concentration and particle composition. Absolute Lp(a)-C levels primarily reflect Lp(a) particle number, whereas particle-normalized indices indicate that cholesterol content per particle increases with apo(a) isoform size. This distinction is not captured by conventional estimation methods and has implications for interpreting Lp(a)-related cardiovascular risk.

A major contribution of this work is the measurement of both the size of the predominant and minor apo(a) isoform along with the application of particle-normalized indices, the Lp(a)-C/Lp(a) molar ratio, and the Lp(a)-C/Lp(a)-apoB mass ratio, to provide an indirect estimate of cholesterol content per particle. Both indices increased progressively across isoform tertiles, more than doubling from the smallest to the largest isoform groups. These relationships were not apparent from Lp(a) molar concentrations alone and reveal that differences in apo(a) size are associated with differences in Lp(a) particle lipid content. Because most patients have two distinct apo(a) isoforms, our findings suggest that the larger isoform is more cholesterol-enriched (and possibly triglyceride-enriched, as indicated by preliminary data in individuals with elevated plasma triglycerides (6)), whereas the smaller isoform contributes more to bulk Lp(a)-C primarily through higher particle number.

Although the inverse relationship between apo(a) isoform size and circulating Lp(a) levels is well known (21), prior studies were unable to determine whether differences in particle composition accompany and potentially modulate this isoform-concentration relationship. Early biochemical analyses suggested that Lp(a) lipid content might not vary with plasma levels (4), but these studies relied on ultracentrifugation or preparative isolation techniques that can alter particle composition and were unable to directly quantify cholesterol on native Lp(a) particles (9, 10, 22). Using a direct immunocapture assay that preserves the native structure of Lp(a), our findings show that apo(a) isoform size is associated not only with particle number but also with the average cholesterol carried per particle, providing new biochemical context for long-standing isoform-dependent variation in Lp(a) levels.

The observation that larger apo(a) isoforms carry more cholesterol per particle suggests potential differences in lipid packaging or remodeling during Lp(a) assembly or circulation. Although direct evidence is not present currently, one possible explanation is that larger apo(a) isoforms sterically alter the conformation of the apoB-100-containing LDL particle to which they are covalently attached, leading to differences in core lipid accommodation that favor greater cholesterol retention. Recent high-resolution structural data of apoB-100 on LDL particles show that apoB-100 forms an extended β-sheet “belt” around the particle, with modular domains accommodating a range of particle sizes (23). These insights support the plausibility that binding of apo(a) isoforms of differing length and conformation could alter apoB-100's configuration and core lipid packing in Lp(a), although direct evidence for such isoform-specific effects is currently lacking. Therefore, these mechanistic interpretations remain speculative and require direct experimental validation.

Alternatively, smaller isoforms may interact differently with hepatic or intravascular remodeling pathways, such as hepatic lipase, endothelial lipase, or cholesteryl ester transfer protein, resulting in differential depletion of cholesteryl esters over time. Because smaller apo(a) isoforms are generally associated with higher particle production rates (24), they may circulate for a longer time (25), allowing more extensive lipid remodeling and progressive cholesterol depletion. Cholesteryl ester transfer protein inhibitors reduce plasma concentrations of Lp(a) but their effects on Lp(a) size and composition are not known (26). Although these mechanisms remain speculative, they provide a plausible framework for isoform-dependent differences in Lp(a) lipid composition.

This study has limitations. First, the blood donors used for method development had relatively low Lp(a) concentrations, which may limit generalizability to individuals with markedly elevated Lp(a). Second, demographic, metabolic, and clinical covariates were not available, and compositional analyses focused solely on cholesterol; therefore, the contributions of triglycerides, phospholipids, and oxidized phospholipids to apo(a)-containing particles remain undefined. These findings provide a biochemical rationale for directly measuring Lp(a)-C in trials of Lp(a)-lowering therapies. Larger cohorts paired with mass-spectrometry-based lipidomics will be needed to delineate metabolic determinants and isoform-dependent patterns of lipid enrichment within Lp(a).

A key next step will be direct compositional analysis of affinity-isolated Lp(a) particles from individuals with extreme apo(a) isoform sizes. Measurement of free and esterified cholesterol in purified Lp(a) using standard biochemical assays will be required to confirm isoform-dependent differences and establish assay reproducibility. Integration of total apoB measurements will also be important to distinguish Lp(a)-associated apoB from LDL particles and to better contextualize Lp(a)-C within total atherogenic particle burden.

In summary, apo(a) isoform size is associated with both Lp(a) particle number and cholesterol content per particle. Direct measurement of Lp(a)-C reveals isoform-dependent compositional heterogeneity that is not captured by conventional estimation approaches. These findings support the use of direct Lp(a)-C measurement in mechanistic and clinical studies and may inform the interpretation of Lp(a)-lowering therapies.

## Data Availability

Data that support the plots within this publication and other findings of this study are available from the corresponding authors upon request.

## Supplemental Data

This article contains supplemental data.

## Conflict of interest

S. M. M. is an employee of Medpace Reference Laboratories and reports consulting roles for Denka. S. T. is a co-inventor and receives royalties on patents held by the University of California on monoclonal antibodies directed to Lp(a) and oxidized phospholipids, is a co-founder and has an equity interest in Oxitope, Kleanthi Diagnostics, and Megaron, and has a dual appointment at UCSD and Ionis Pharmaceuticals.

## Supplemental Data

Supplementarl Figures S1–S3
