# Supplementary material for: Direct Lp(a)-C measurements provide evidence for Apo(a) isoform-dependent cholesterol composition of Lp(a)
Source: J Lipid Res. 2026 Apr 27;67(6):101051. doi: 10.1016/j.jlr.2026.101051 (PMC13226922; doi:10.1016/j.jlr.2026.101051)

**Supplement**

**Direct Lp(a)-C Measurements Provide Evidence for Apo(a) Isoform-Dependent Cholesterol Composition of Lp(a)**

# Sotirios Tsimikas, Santica M. Marcovina

**Figure S1.** Relationship of predominant apo(a) isoform size to Lp(a) parameters. Scatterplots show Spearman correlations between predominant apo(a) isoform KIV repeat number and the Lp(a)-C/Lp(a)-apoB mass ratio.

**Figure S2.** Relationship of minor apo(a) isoform size to Lp(a) parameters. Scatterplots show Spearman correlations between minor apo(a) isoform KIV repeat number and the Lp(a)-C/Lp(a)-apoB mass ratio.

**Figure S3.**Lp(a) composition across tertiles of apo(a) isoform size. (A-B) Lp(a)-C/Lp(a)-apoB ratios across the same tertiles for the predominant (A) and minor (B) isoforms.


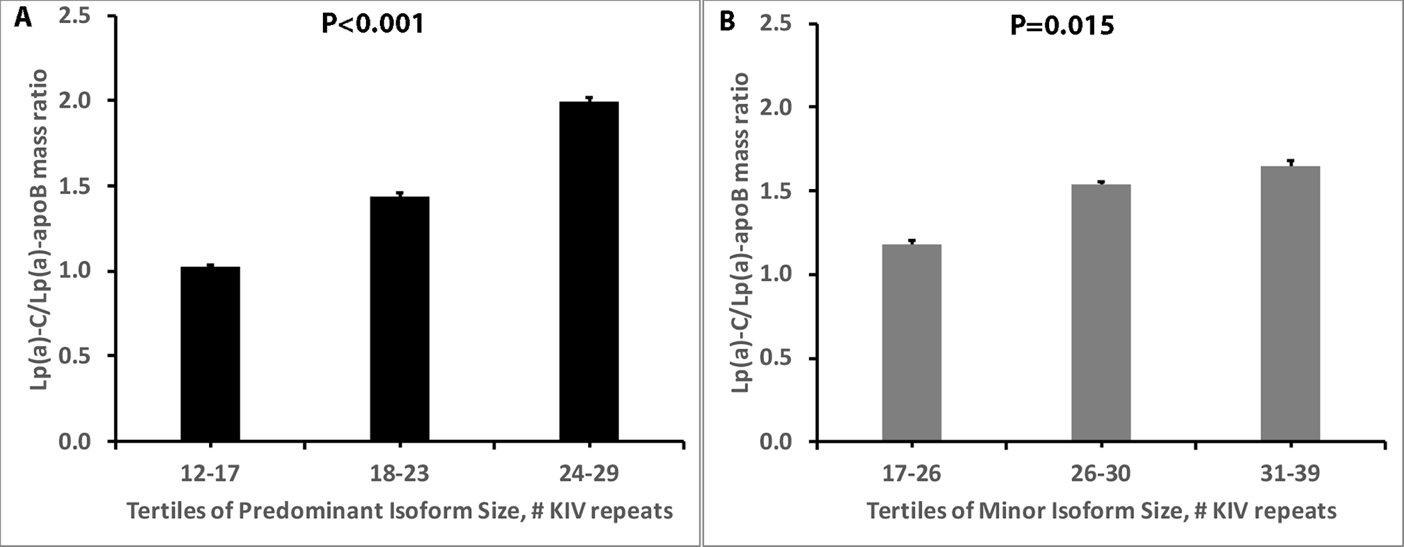

Supplement: Supplementarl Figures S1–S3 [file mmc1.docx]
